# Can laboratory x-ray virtual histology provide intraoperative 3D tumor resection margin assessment?

**DOI:** 10.1117/1.JMI.9.3.031503

**Published:** 2022-02-07

**Authors:** William Twengström, Carlos F. Moro, Jenny Romell, Jakob C. Larsson, Ernesto Sparrelid, Mikael Björnstedt, Hans M. Hertz

**Affiliations:** a KTH/Albanova, Department of Applied Physics, Stockholm, Sweden; b Karolinska University Hospital, Department of Clinical Pathology and Cancer Diagnostics, Stockholm, Sweden; c Karolinska University Hospital Huddinge, Karolinska Institutet, Division of Pathology F46, Department of Laboratory Medicine, Stockholm, Sweden; d Karolinska Institutet, Division of Surgery, Department of Clinical Science, Intervention and Technology, Stockholm, Sweden

**Keywords:** human resection margin assessment, phase-contrast tomography, propagation-based imaging

## Abstract

**Purpose:**

Surgery is an essential part of the curative plan for most patients affected with solid tumors. The outcome of such surgery, e.g., recurrence rates and ultimately patient survival, depends on several factors where the resection margin is of key importance. Presently, the resection margin is assessed by classical histology, which is time-consuming (several days), destructive, and basically only gives two-dimensional information. Clearly, it would be advantageous if immediate feedback on tumor extension in all three dimensions were available to the surgeon intraoperatively.

**Approach:**

We investigate a laboratory propagation-based phase-contrast x-ray computed tomography system that provides the resolution, the contrast, and, potentially, the speed for this purpose. The system relies on a liquid-metal jet microfocus source and a scintillator-coated CMOS detector. Our study is performed on paraffin-embedded non-stained samples of human pancreatic neuroendocrine tumors, liver intrahepatic cholangiocarcinoma, and pancreatic serous cystic neoplasm (benign).

**Results:**

We observe tumors with distinct and sharp edges having cellular resolution (∼10  μm) as well as many assisting histological landmarks, allowing for resection margin assessment. All x-ray data are compared with classical histology. The agreement is excellent.

**Conclusion:**

We conclude that the method has potential for intraoperative three-dimensional virtual histology.

## 1 Introduction

Surgery is an essential part of the curative plan for most patients affected with solid tumors. The outcome of such tumor surgery, e.g., recurrence rates and ultimately patient survival, depends on several factors where the resection margin is of key importance. In the present paper, we show that laboratory-source-based phase-contrast x-ray imaging has potential to provide rapid intraoperative information of the resection margin.

The resection margin, or surgical margin, is the margin of apparently non-tumorous tissue around a tumor that has been surgically removed. Numerous of studies have consistently shown that margin status is a key indicator for patient outcome.^1^^–^^9^ However, the optimal minimal distance between the cancer cells and the resection margin is still debated. Often it is preferable to keep the margin as small as possible, e.g., for cosmetic reasons (e.g., breast and skin) or to preserve organ-function (e.g., liver and kidney). Still, no malignant growth should extend past the resection margin, as this would indicate incomplete surgical removal of the tumor. For this purpose, all surgical oncology is followed by a pathological assessment not only of the tumor itself but also of the removed surrounding tissues and in particular the resection margin. This is presently a time-consuming and largely manual process involving many steps, starting with formalin fixation, dehydration, and paraffin embedding, followed by classical multistep histology. It typically takes several days or a week before the surgeon receives the pathology report. If the resection margin turns out insufficient, this may increase the risk for local recurrence, seriously compromising patient outcome. Furthermore, due to the present process of cutting in 3- to 5-mm slices, the pathology risks missing small groups of cells inside the slices.

It clearly would be advantageous if the surgeon could get immediate feedback from the pathologist intraoperatively. This would require rapid image acquisition and data processing near the surgical suite on samples with minimum preparation. Such fast feedback would allow corrective action for complete surgical tumor removal and improved patient outcome. Furthermore, it would be advantageous if the pathological assessment could be done in full three dimensions with cellular resolution, to capture all cellular-sized pathological features.

Present 3D imaging methods (e.g., computed tomography [CT], magnetic resonance imaging [MRI], positron emission tomography [PET], and ultrasound [US]) do not provide sufficient resolution and/or contrast for this purpose. However, phase-contrast x-ray imaging^10^^–^^12^ can provide rapid and high-resolution two-dimensional (2D) and 3D imaging on unstained soft-tissue samples. There are several phase-contrast imaging methods suitable for synchrotron sources, but only grating-based imaging (GBI) and propagation-based imaging (PBI) have been widely used with laboratory sources,^13^ which are necessary for locating the system close to the operating room. Although the intrinsic quantitative nature of GBI is an advantage, the extra optical elements (gratings) and multistep exposure result in longer exposure times than for PBI, with its free-space propagation and single exposure. There are only few comparisons on observable detail versus exposure time for the two methods, but for imaging gas-filled structures (like ${\mathrm{CO}}_{2}$-filled blood vessels or air-filled lung alveoli), the necessary dose for observing sub-50-μm structures may differ by a factor of 10 in favor of PBI.^14^ Given that laboratory systems are typically limited by source power, this factor 10 difference also directly translates into a shorter exposure time. For the intraoperative pathological assessment of resection margins, exposure time will be of utmost importance. Therefore, PBI is the preferred method.

X-ray phase contrast imaging has been suggested as an alternative to conventional histology on excised samples.^15^^–^^21^ The advantages include speed (both in preparation and acquisition), isotropic 3D resolution (with thinner effective slicing), and simplified and less destructive sample preparation, all important properties for resection margin assessment. At synchrotron radiation sources such virtual x-ray histology has been demonstrated on unstained tissue with sufficient cellular resolution and contrast, e.g., on rat testicle,^15^ mouse kidney,^21^ and mm-sized punches of human brain^19^ and breast.^20^ Baran et al.^20^ sought to provide tumor demarcation. However, only few laboratory-source virtual-histology experiments have demonstrated the necessary (preferably cellular) resolution and contrast in unstained tissue: zebrafish,^17^ human coronary arteries,^18^ and human brain.^19^ For clinical application, the local access of a laboratory system close to the operating suite is of key importance. Finally, we note that several studies have used conventional absorption x-ray microCT systems for histology, but they typically require stained samples to reach the necessary high resolution,^22^^,^^23^ and high-contrast breast specimens have been examined at lower resolution with a laboratory phase-contrast system.^24^

In the present paper, we show that laboratory propagation-based phase-contrast x-ray tomography of unstained resected tissue has the proper properties for rapid and high-resolution 3D assessment of the resection margins. We demonstrate our method on paraffin-embedded samples with two malignant tumors (pancreatic neuroendocrine tumor and liver intrahepatic cholangiocarcinoma) and one benign tumor (pancreatic serous cystic neoplasm) and compare with classical histology for verification. Our system relies on a small-spot high-power liquid-metal-jet x-ray tube, enabling propagation-based phase-contrast x-ray imaging with high spatial resolution (cellular) and adequate exposure times. Tailored algorithms are essential for the image reconstruction. Finally, we discuss the potential of the method for intraoperative resection margin assessment.

For completeness, we note that in tumor surgery, intraoperative histology can be performed with cryofrozen thin sections with or without x-ray imaging.^25^^–^^27^ Unfortunately, the freezing induces many tissue artifacts, making results difficult to interpret on these normally small samples. The x-ray imaging relies on absorption only, making resection margin assessment difficult. We also note that visible-light methods are employed for intraoperative fluorescence diagnostics, albeit with great difficulty due to low light levels.^28^

## 2 Materials and Methods

### 2.1 Propagation-Based X-Ray Phase-Contrast Tomography Arrangement

**Fig. 1:**
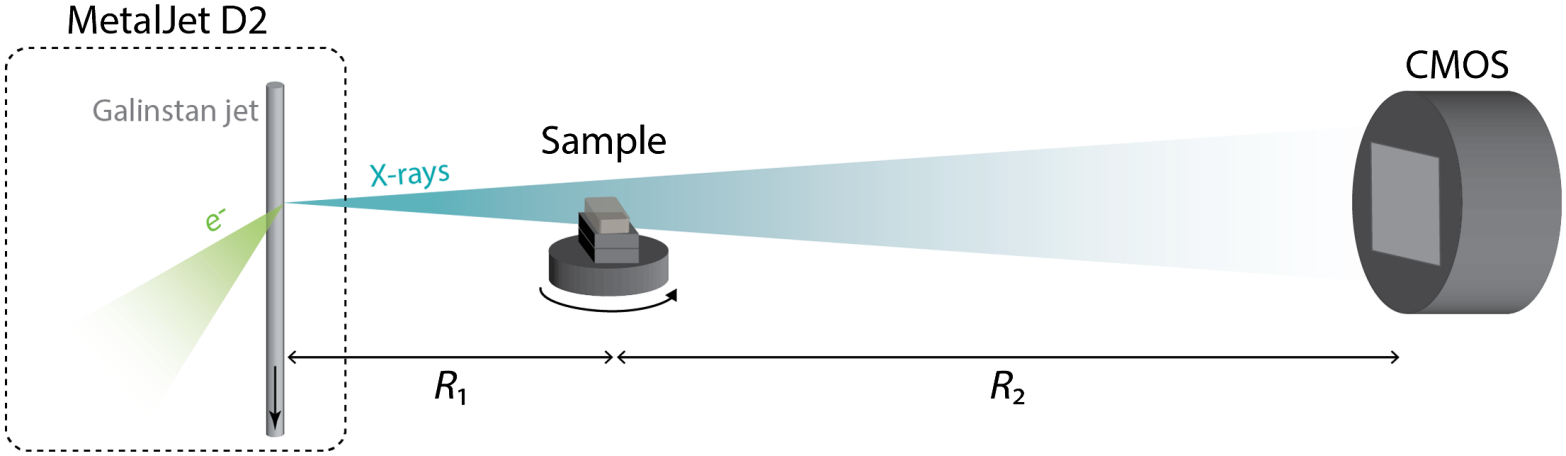
Propagation-based phase-contrast x-ray imaging of tissue samples. The microfocus liquid-metal-jet x-ray source illuminates the sample, and the image is detected by a CMOS detector. $R_{1}$ and $R_{2}$ determine phase contrast and magnification.

Section 1 provides an overview of phase-contrast tomography. Figure 1 shows the experimental arrangement, described in detail in Refs. 17 and 18. It relies on a liquid-metal-jet microfocus x-ray source, a high-resolution scintillator coupled x-ray camera, and the sample on a rotating stage. We use a MetalJet D2 (Excillum AB, Sweden) operated at 50 to 70 kV and 71 to 129 W, focused to a 10×40 or 15×60  μm spot on the Galinstan jet. The rotation stage was a Newport URS50BCC (Newport, California) and the detector a Photonic Science (Photonic Science, UK) CMOS detector with 4096×4096  pixels with a pitch of 9  μm, fiber-optically coupled (1:1) to a 10  μm gadolinium oxysulfide scintillator. The detector point-spread function had a full-width half-maximum of 22  μm.

The source-sample distance ($R_{1}$) and the sample-detector distance ($R_{2}$) define the geometry. The magnification of the sample to the detector is $M=(R_{1}+R_{2})/R_{1}$ enabling imaging resolutions better than the detector resolution. The phase contrast is achieved from an effective propagation distance $z_{\mathrm{eff}}=R_{1}R_{2}/(R_{1}+R_{2})$, due to the divergent cone beam. M and $z_{\mathrm{eff}}$ can be set independently by changing $R_{1}$ and $R_{2}$ and were adjusted to obtain good phase contrast and signal-to-noise ratio^29^ while still keeping the full width of the sample in the field of view. In the present study, we optimize the system to allow for detection with cellular resolution, i.e., the 10-μm range, while still maintaining reasonable exposure times.^29^

### 2.2 Samples and Sample Preparation

**Fig. 2:**
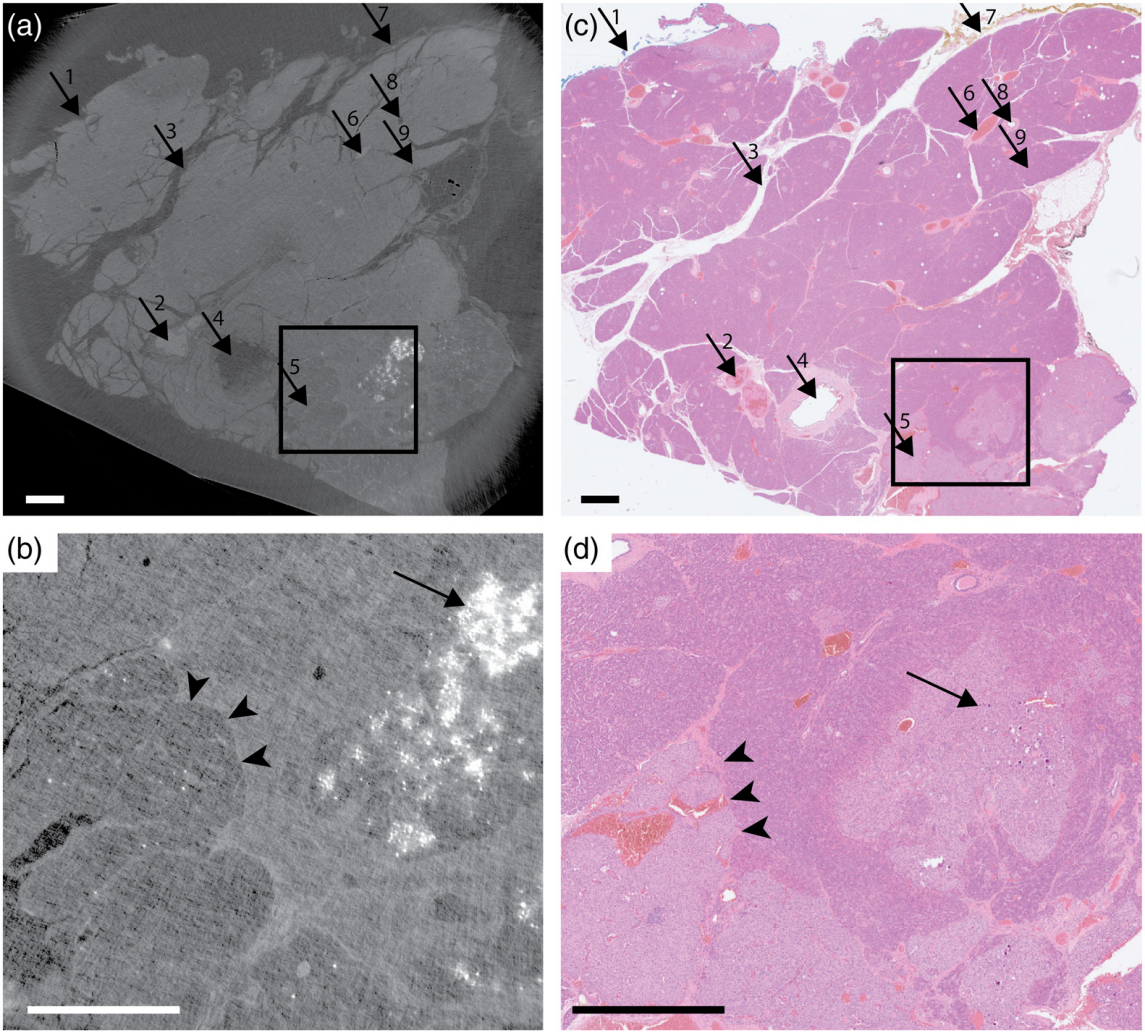
Neuroendocrine tumor in pancreas: (a) the x-ray tomography and (c) the histology. Arrows: 1, posterior resection margin; 2, middle size blood vessel; 3, interlobular septum; 4, large size, main pancreatic duct; 5, neuroendocrine tumor; 6, small size blood vessel; 7, inferior resection margin; 8, small size pancreatic duct; and 9, pancreatic lobuli. The tumor (5) has lower density than surrounding tissue and can clearly be identified with both methods. (b), (d) Magnified images as indicated by the boxes in (a) and (c), respectively. Arrowheads indicate the sharp cellular demarcation between tumorous and healthy tissue, clearly visible in both the x-ray tomography (assisted by a slight residual phase edge enhancement) and the histology. Long arrow indicates microcalcifications in tumorous tissue, clearly visible in the x-ray tomography. The microcalcifications are still visible in the histology, but not as apparent as in the x-ray tomography. All scale bars are 1 mm (Video 1, MP4, 5277 kB [URL: https://doi.org/10.1117/1.JMI.9.3.031503.1]).

**Fig. 3:**
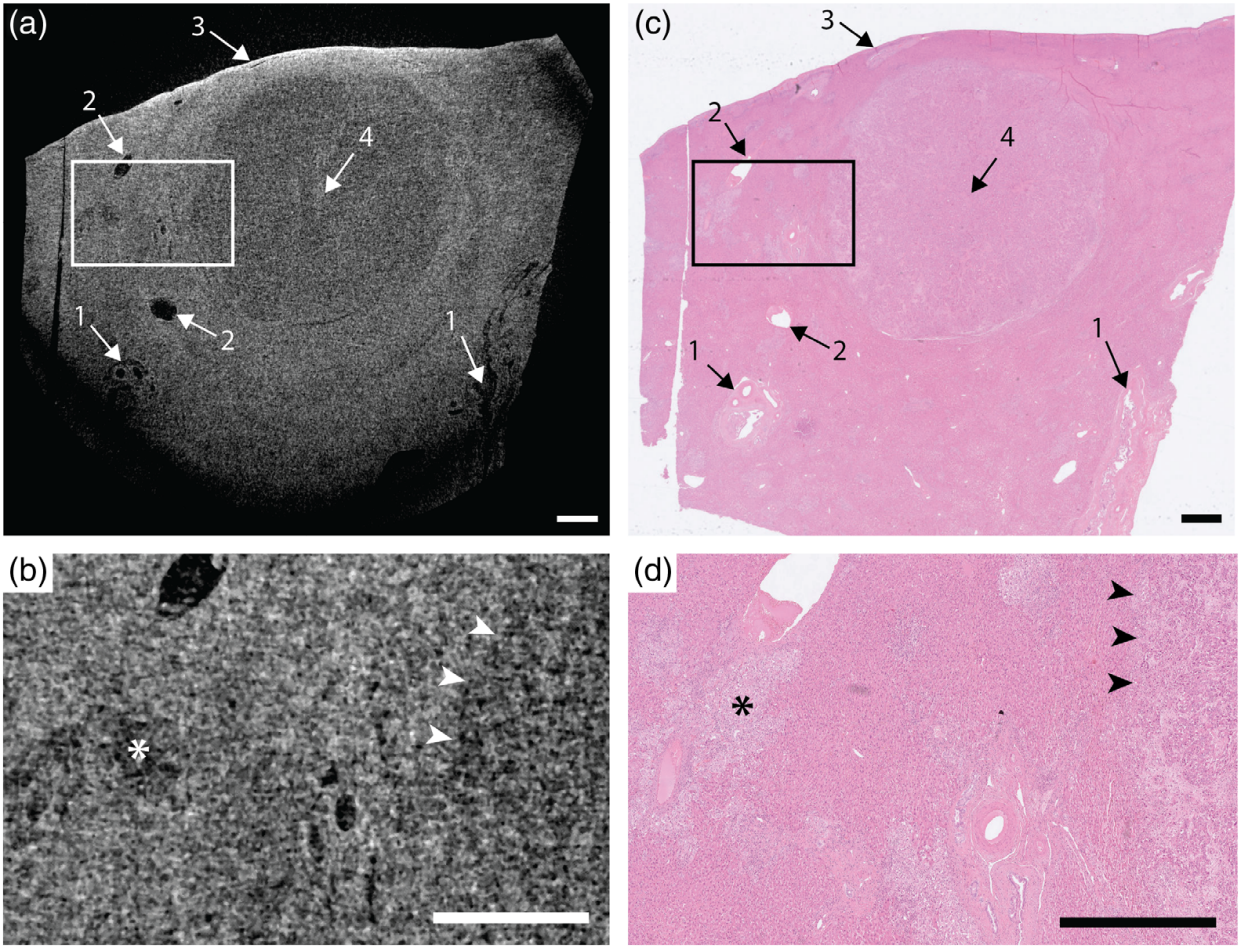
Intrahepatic cholangiocarcinoma in liver: (a) x-ray tomography and (c) histology. Arrows: 1, portal triads; 2, central veins; 3, liver capsule; and 4, cholangiocarcinoma. The nodular tumor has lower density than surrounding tissue and can clearly be identified with both methods. (b), (d) A detailed view of the tumor border (marked with arrowheads) in tomography and histology, respectively. The asterisk (*) marks a separate (satellite) small region of tumorous tissue. All scale bars are 1 mm (Video 2, MP4, 7789 kB [URL: https://doi.org/10.1117/1.JMI.9.3.031503.2]).

**Fig. 4:**
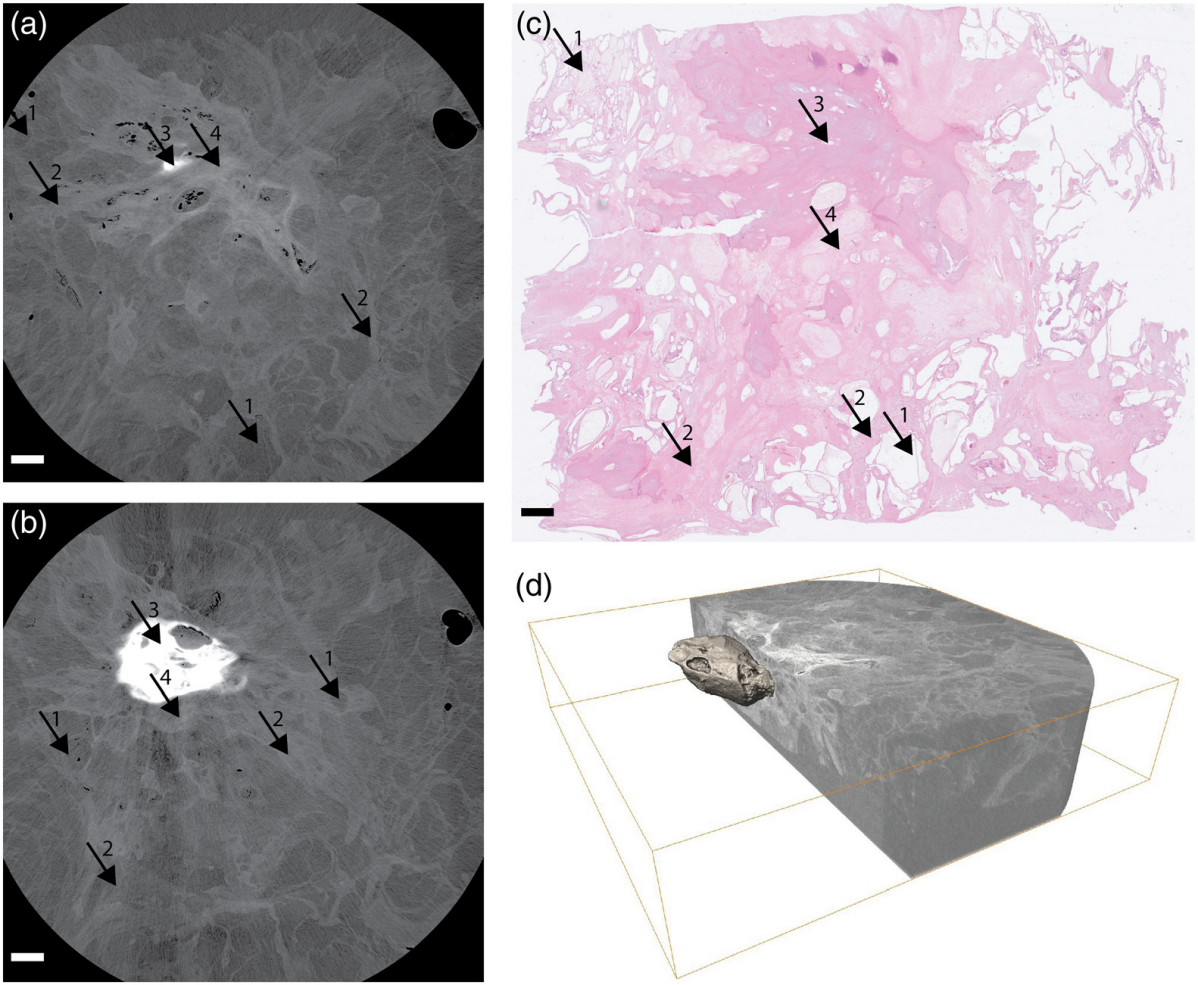
Serous cystic neoplasm in pancreas: (a) the x-ray tomography closely below the histology, where the high-density tumor starts to be visible. In panel (b), a slice 500  μm below (a), the high-density calcified core of the tumor is clearly visible. (c) The histology: arrows: 1, small cysts; 2, fibrous septum; 3, central scar, calcified region; and 4, central scar, fibrous region. (d) A 3D image of the central part of the tumor, showing the extent of the calcified core and why it is barely visible in the classical histology slice. All scale bars are 1 mm (Video 3, MP4, 7366 kB [URL: https://doi.org/10.1117/1.JMI.9.3.031503.3]).

Our method is tested on unstained paraffin-embedded standard histological tissue samples from a biobank, typically $2\times 3\times 0.5\;{\mathrm{cm}}^{3}$. We show examples of a pancreatic neuroendocrine tumor (Fig. 2), liver intrahepatic cholangiocarcinoma (Fig. 3), and a pancreatic serous cystic neoplasm (Fig. 4, benign). Directly after surgery, the entire specimen was fixed in formalin ∼3 days. After grossing of the formalin-fixed surgical specimen, tissue samples were processed for histology using a vacuum infiltration processor, according to the standard operating procedure: formalin fixation (∼0.5  h), ethanol dehydration (typically seven steps of different ethanol concentrations, 1 to 3 h each), xylen intermediary (typically two steps 1 to 2 h each) and, finally, paraffin impregnation (typically four steps, 1 h each). Afterward, tissue samples were embedded in paraffin. All samples were pseudoanonymized and handled in accordance with the ethical permit (Regionala Etikprövningsnämnden in Stockholm 2019-00583). In total, 16 tumor samples from 10 patients operated at Karolinska University Hospital were investigated.

### 2.3 Phase Contrast X-Ray Imaging

The source-sample-distance was 25 to 28 cm, and the source-detector-distance 60 to 64 cm. Thus the magnification was M=2.3 to 2.4 resulting in a 3.8- to 3.9-μm pixel size in the sample plane. The detector-limited resolution in the sample plane is 9  μm. For each tomographic dataset, 2400 to 3600 projections were recorded over 360 deg with an exposure time of 2.5 to 4.5 s per projection. The total exposure time was 100 to 180 min.

### 2.4 Image Processing

The projection images were dark- and flat-field corrected, and phase retrieved using Paganin’s method,^30^ with $\delta /\mu =2\times 10^{-8}$ and λ=0.124  nm. Multiple algorithms for ring artifact reduction, destriping, and denoising where employed.^31^^–^^35^ Other algorithms may be applicable. The phase-retrieved images were tomographically reconstructed using the FDK cone-beam algorithm from Octopus Reconstruction (TESCAN, Gent, Belgium). A 3×3×3 median filter was applied to the reconstructed volume images to reduce noise. Gray scales have been adjusted in all images. ImageJ (National Institutes of Health, Bethesda, Maryland) and Amira (Thermo Fisher Scientific, Waltham, Massachusetts) were used for postprocessing and volume renderings.

### 2.5 Comparative Histology

This study was performed as a retrospective study using material from a biobank. Thus routine clinical histopathology procedures to obtain a section of 4  μm and standard hematoxylin and eosin staining were performed before the x-ray imaging. This study utilizes the original histopathology slides for comparison, and consequently the section imaged in histopathology had already been removed from the surface of the paraffin-embedded tissue sample when it was imaged with x-ray phase contrast.

## 3 Results

### 3.1 Neuroendocrine Tumor in Pancreas

Figure 2(a) shows the propagation-based phase-contrast x-ray tomography in a tomographic slice close to (<0.5  mm) the top layer of the paraffin-embedded slice. The sample is $2\times 3\;{\mathrm{cm}}^{2}$ and the tomography is based on 2401 projections acquired with a total exposure time of 180 min. We note the characteristic lobular histological architecture of normal pancreatic parenchyma where the lobuli are separated by septa containing different sized ducts and blood vessels with erythrocytes in lumen. The neuroendocrine tumor in the bottom right of Fig. 2(a) is darker due to its lower density than the surrounding tissue [Fig. 2(b)]. Intratumoral microcalcifications are clearly visible as white, due to their higher absorption. The phase-contrast modality provides sharp and distinct edges [Fig. 2(b), arrowheads] between regions of different refractive indices, enabling assessment of the tumor and the resection margin with a resolution down to ∼10  μm. We note that some residual edge enhancement is present. The “video_Fig2” shows the full 3D stack.

Figures 2(c) and 2(d) show the top-layer histology from the same paraffin slice, with hematoxylin and eosin staining. Note the similarity with the x-ray image, although they are not identical due to slightly different vertical positions of the tomographic slice and the histological slices. We observe the tumor and its margins with clarity, confirming the results of the x-ray tomography.

### 3.2 Intrahepatic Cholangiocarcinoma in Liver

Figure 3(a) shows the propagation-based phase-contrast x-ray tomography, in a tomographic slice close to (<0.5  mm) the top layer of the paraffin-embedded slice. The sample is $2\times 3\;{\mathrm{cm}}^{2}$ and the tomography is based on 2400 projections acquired with a total exposure time of 100 min. Like the pancreatic neuroendocrine tumor, the intrahepatic cholangiocarcinoma has lower density than its surroundings and thus appears darker in the tomography image. The edge of the tumor is not as sharp as in the pancreas tumor shown above, but still clearly distinguishable. We also note different sized portal triads, central veins (both appearing black), and the liver capsule (white). The marked region is shown magnified in Fig. 3(b), where the border of the tumor is indicated with arrowheads. A low-density structure (*) is identified as a small (200 to 300  μm) separate satellite region of tumorous tissue outside the major tumor. The “video_Fig3” shows the full 3D stack.

Figures 3(c) and 3(d) show the histology of approximately the same part of the sample, stained with hematoxylin and eosin.

### 3.3 Serous Cystic Neoplasm in Pancreas

Figure 4 shows this benign tumor with abundant fibrous tissue and a calcified core. Figures 4(a) and 4(b) show the phase-contrast tomographic reconstruction at two vertical positions in the sample, close to the top and 500  μm down the stack, respectively. The sample is $2\times 3\times 0.5\;{\mathrm{cm}}^{3}$ and the tomography is based on 3600 projections, with total exposure time of 150 min. Figure 4(c) shows the top-layer histology. Note the characteristic sponge-like architecture with numerous small sized, thin-walled cysts, and a partially calcified central fibrous scar from which fibrous septa radiate toward the periphery. Finally, Fig. 4(d) shows an excerpt from a video clearly showing that central calcified core of the tumor is thinner than the histology slice. This illustrates the virtue of having 3D virtual histology with higher vertical spatial resolution than the classical histology 3- to 5-mm slicing. The “video_Fig4” shows the full 3D stack.

## 4 Discussion

We show that laboratory-source-based phase-contrast x-ray tomography has potential to provide the 3D virtual x-ray histology necessary for intraoperative 3D assessment of the resection margin in cancer surgery. The method is demonstrated on paraffin-embedded samples of human liver and pancreas tumors and compared with classical histology. We show that this can be done with the high (cellular) resolution on unstained samples to allow for intraoperative clinical application.

Presently, the image acquisition takes 1.5 to 3 h. This is too long for intraoperative use. However, the source power used in the present experiments (70 to 130 W) is 5 to 10× below that of the best state-of-the-art microfocus sources (see Ref. 36). Such a higher-power source would bring down the exposure time to 10 to 20 min, a feasible time for intraoperative application of the method.

The next step will be to test the method on fresh tumor samples. Here spatial fixation during the data acquisition will become a challenge since also minute (few microns) movements will lower the edge contrast, resulting in a decreased definition and reduced visibility of the tumors. In addition, it cannot be excluded that some (edge) contrast between tumor and normal tissue is due to differences in shrinking in the dehydration steps of the sample preparation, despite that the process is designed to minimize such differential shrinking. Thus the contrast in fresh or rapidly fixed tissues may need further investigation and optimization. In addition, this experiment will require a larger-diameter detector to allow for imaging full size of the samples while keeping distances long enough for resolution and contrast. The quantum efficiency of the detector can and should be increased but still larger tumors will need to be cut into suitable (few cm) pieces to allow for the 25-kV radiation to penetrate.

In summary, we have demonstrated a compact laboratory system (1×1×2  m) that can be placed close to the operating suit with potential to allow 3D intraoperative resection margin assessment using phase-contrast x-ray tomography. The combined time for preparation, exposure, and analysis is foreseen to approach 20 to 30 min, which is consistent with intraoperative use. Both for hepatobiliary and pancreatic surgery (but also for many other tumor types), it would of great clinical value if intraoperative tumor margins could be promptly assessed with high accuracy. Thus, such x-ray diagnostics hold promise to significantly enhance outcomes in surgical oncology.
